# A comparison of the duration of post-treatment protection of artemether-lumefantrine, dihydroartemisinin-piperaquine and artesunate-amodiaquine for the treatment of uncomplicated malaria

**DOI:** 10.1186/1475-2875-13-S1-P19

**Published:** 2014-09-22

**Authors:** Michael T Bretscher, Jamie T Griffin, Pierre Hugo, Mark Baker, Azra Ghani, Lucy Okell

**Affiliations:** 1 Imperial College London, London, UK; 2 Medicines for Malaria Venture (MMV), Geneva, Switzerland

## Background

Five artemisinin combination therapies (ACTs) are currently recommended for treatment of uncomplicated malaria in Africa. Whilst the artemisinin component has a short half-life, the partner drugs give rise to differing durations of post-treatment prophylaxis, protecting from re-infection and reducing transmission. We compared the duration of post-treatment prophylaxis of artemether-lumefantrine (AL), dihydroartemisinin-piperaquine (DHA-PPQ) and artemisinine-amodiaquine (AS-AQ).

## Materials and methods

We pooled the results from analyses of 6 trials (1651 individuals) of AL versus DHA-PPQ and from 18 trials (5060 individuals) of AL versus AS-AQ. The time to re-infection after treatment was used to estimate the duration of post-treatment protection accounting for the variation in transmission intensity between settings. This duration was then used in a mathematical model of malaria transmission to estimate the comparative public health impact of each drug used for 1st-line treatment in low, medium and high transmission settings.

## Results

We estimated a mean duration of post-treatment protection of 10.7 days (95% CI: 10.2-11.2) and 13.8 days (95% CI: 10.2-22.8) for AL from the two analyses, 29.4 days (95% CI: 16.4-48.8) for DHA-PPQ, and 11.8 days (95% CI: 11.0-12.5) for ASAQ. There was no substantial difference in the public health impact of using AL versus AS-AQ. Use of DHA-PPQ for 1st-line treatment averted 8.3 additional cases per 1,000 people (0.8% of all cases) over 5 years compared to AL or AS-AQ. This protective effect was stronger in areas of high transmission as well as in seasonal settings.

## Conclusions

While it is important to maintain a diverse set of first-line treatments for uncomplicated malaria, our results suggest that partner drugs with a longer half-life could be beneficial in reducing repeat episodes and onward transmission, particularly in high and seasonal transmission areas.

